# Establishment of a Pediatric Surgical Unit at a University Hospital in Eastern Africa

**DOI:** 10.3390/children8030244

**Published:** 2021-03-22

**Authors:** Jochen Hubertus, Gersam Abera, Abraham Haileamlak, Matthias Siebeck, Dietrich von Schweinitz, Ferdinand Wagner, Rosa Eckle, Lucas Wessel, Laura Ritz, Mircia Aurel Ardelean, Kristina Becker, Alemu Seifu

**Affiliations:** 1 Department of Pediatric Surgery, Dr. von Hauner Children’s Hospital, LMU University Hospital, 80337 Munich, Germany; dietrich.schweinitz@med.uni-muenchen.de (D.v.S.); ferdinand.wagner@med.uni-muenchen.de (F.W.); laura.ritz@med.uni-muenchen.de (L.R.); kristina.becker@med.uni-muenchen.de (K.B.); 2 Department of Surgery and Child Health, Jimma University Hospital, Federal Ministry of Health, Jimma 47, Ethiopia; gersamabera@yahoo.com (G.A.); asratab@yahoo.com (A.H.); alemu_seifu@yahoo.com (A.S.); 3 Department of General, Visceral and Transplantation Surgery, LMU University Hospital, 80337 Munich, Germany; matthias.siebeck@med.uni-muenchen.de; 4 JoynCoop, Strategy Consultancy and Co-Creation for Human Development, 80538 Munich, Germany; r.eckle@joyn-coop.com; 5 Department of Pediatric Surgery, Medical Faculty of Heidelberg, University of Mannheim, 68135 Mannheim, Germany; lucas.wessel@medma.uni-heidelberg.de; 6 Department of Pediatric Surgery, University of Salzburg, 5020 Salzburg, Austria; m.ardelean@salk.at

**Keywords:** Ethiopia, pediatric surgery, development cooperation, specialized care

## Abstract

Introduction: Ethiopia is a rapidly developing country in Eastern Africa. In total, 43.2% of the population are younger than 15. In contrast, until a few years ago, pediatric surgery was only available in Addis Ababa. Now, Ethiopia is making great efforts to improve the care of children who require surgery. JimmaChild was established to set up a pediatric surgery in Jimma. Material and methods: JimmaChild developed from a scientific collaboration between Jimma University (JU) and Ludwig-Maximilians-University. The project was developed and realized by Ethiopian and German colleagues. A curriculum was written for this purpose. The pediatric surgical training of the fellows was carried out on-site by German pediatric surgeons. Results: A new pediatric surgery was established at JU with its own operating room, ward, and staff. After two and a half years, two fellows completed their final examinations as pediatric surgeons. Among others, 850 elective surgeries were performed, 82% assisted by the German colleagues. The German colleagues rated the preparation for the trip, the on-site support, and the professional progress of the fellows mostly as good to very good. Reported problems in the program flow were also recognized and solved in part. Conclusions: The best possible integration of the project into existing structures was achieved by close cooperation of Ethiopian and German colleagues during the project development. Problems were identified and addressed early on by external monitoring. As the project responsibility was mainly with the Ethiopian colleagues, a department was created that now exists independently of external funding and trains its own fellows.

## 1. Introduction

Ethiopia is a country in East Africa with a very young population. The birth rate is 36 births/1000 inhabitants. In total, 43.2% of the population are younger than 15 [1]. In contrast, by 2016, there were only five trained pediatric surgeons in Addis Ababa, resulting in insufficient pediatric surgical care in most parts of Ethiopia. The majority of sick children were initially treated by general surgeons. This approach is similar to Calisti et al.’s concept of involving general surgeons in the care of children [2]. However, this approach does have significant limitations, so that patients with complex conditions used to be referred to the Tikur Anbessa Hospital in Addis Ababa. Elective pediatric surgeries at this hospital had to wait many months. This reflects the problem that, in recent years, while recognizing the central role of surgery for global health, pediatric surgery has played a subordinate role [3].

**Figure 1:**
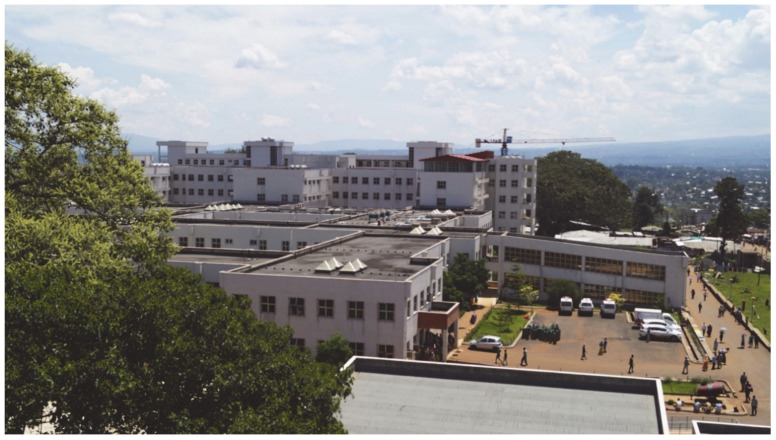
A bird’s eye view of Jimma University Teaching Hospital. The building was ready to be opened in 2016.

In 2014, the Jimma University (JU) in Oromia, Ethiopia, in cooperation with the Department of Pediatric Surgery at the Dr. von Hauner Children’s Hospital in Munich, Germany, set out to establish a pediatric surgical unit at the JU Hospital (Figure 1).

The aim was to have a pediatric surgical unit that offers modern patient care within the framework of local conditions, independently of external donors, and capable of training future fellows. As such, the JimmaChild project was born.

We describe the fruitful cooperation and the success we had. However, we also focus on the difficulties we encountered and the problems we faced during the project. 

## 2. Materials and Methods

JimmaChild emerged from a long-standing cooperation between the JU and the Ludwig-Maximilians University (LMU). Within this framework was a lively academic exchange within various disciplines, expanded to include pediatric surgery in 2014. Bilateral exploratory meetings were held to evaluate the conditions in Jimma.

### 2.1. Project Development

The future pediatric surgery should be able to exist independently of external donors, enable the training of future pediatric surgeons, and ensure the care of even critically ill children who require surgery. The challenge was to act according to the current state of science but with consideration of local conditions. For this purpose, it was decided that (I) a pediatric surgical curriculum would be developed and adopted and (II) two Ethiopian general surgeons would be trained as pediatric surgeons.

Central to the project was a clear understanding that the Ethiopian and German colleagues would actively collaborate on the development.

The curriculum focused primarily on teaching pediatric surgical skills. Other aspects such as research activities, annual evaluations, continuing education, and internships were also addressed. Final examination would consist of a local exam followed by the board examination of the College of Surgeons of East, Central, and Southern Africa (COSECSA).

Accordingly, two surgeons—referred to henceforth as fellows—underwent pediatric surgical training. It was decided that the training would be carried out primarily at JU, to treat complex diseases at a high level but under local conditions. They were taught surgical skills and trained in leadership functions.

Through the German Society for Pediatric Surgery (DGKCH), experienced German pediatric surgeons were invited to apply for JimmaChild. Criteria for participation were a minimum of 10 years of work experience and special expertise in a subfield of pediatric surgery. Six times a year, selected German doctors traveled to Jimma for four weeks. Their task was to teach the Ethiopian fellows the pediatric surgical techniques. In addition, the Ethiopian surgeons each traveled to Munich for two four-week periods each to observe the pediatric program and gain insight into the German health care system. 

### 2.2. Curriculum

The pediatric surgical curriculum was developed mainly by the JU team and edited by the German participants. The curricula from Tikur Anbessa Hospital and the German curriculum served as a foundation and were adapted to local conditions. Where equipment was not available at JU, training content would be omitted. This particularly affected the field of minimal invasive surgery. Other elements, such as research services, were able to be adopted. The final curriculum, designed to be completed in two and a half years, presupposes that a candidate had completed specialist training as a general surgeon (see Supplementary File S1).

### 2.3. Resources

During the project preparation phase, it became apparent that even complex diseases could be treated at the JU Hospital, except that their instrumentation was not suitable for pediatric surgery. Therefore, three pediatric instrument trays were donated to JU. In addition, magnifying glasses and technical literature were made available to the two fellows. No additional equipment was purchased.

### 2.4. Preparatory Workshop

In several preparatory workshops, the German colleagues were informed about various aspects of their trip. (I) They received details about their flights and what to expect regarding hotel, food, and hygiene. (II) They learned about their assignment on-site, which mainly consisted of conducting the pediatric surgical training by assisting in the operations whenever possible. (III) They also learned about the specifics of medical care at the JU Hospital with a focus on hygienic conditions, limited diagnostic and therapeutic options, and the high infant mortality rate. (IV) It was clearly conveyed that only operations may be performed in which the German colleague feels confident. If not, the patient is to continue to receive only primary care in Jimma and be transferred to the Tikur Anbessa Hospital. (V) The German surgeons were asked to integrate into the host structure rather than taking on the role of the leader. It was made clear that the Ethiopian fellows were the people responsible for implementing JimmaChild. When a problem was noticed by someone from the German team, they were to discuss it with the fellows to find the best approach to a solution. The implementation was then left to the discretion of the hosts.

### 2.5. Additional Opportunities

In addition to the specialist training on-site, the Ethiopian surgeons were each invited to Germany for two four-week observerships. This led to a strong professional and intercultural exchange. One of the fellows was also invited to travel to the United States of America twice for four weeks each time. During the first visit, a management course was offered specifically for project managers in sub-Saharan Africa. During the second stay, he completed an observership at one of the most renowned clinics worldwide in the treatment of anorectal malformations.

### 2.6. National Cooperation

JimmaChild has always seen itself as only one part of a national plan to improve the care of surgical ill children. Tikur Anbessa Hospital offers a highly experienced clinic with a full spectrum of pediatric surgery. Therefore, it was crucial that the team at Tikur Anbessa Hospital was available as a medical backup.

### 2.7. Monitoring

External monitoring was carried out to identify problematic developments at an early stage. For this purpose, the German colleagues had to fill out a questionnaire after their stay (see Supplementary File S2). Questions about their stay and the professional development of the fellows were asked. The questionnaire included free-text questions and multiple-choice questions (Likert scale ranging from very good (1) to poor (5)). These responses enabled the team to react promptly to problematic developments and the questionnaires were used to educate the next German colleagues about the current status of the project. For an objective view, monitoring was carried out by the consulting company JoynCoop.

### 2.8. Final Examination

The final examination consisted of two components: a multiple-choice examination and a clinical examination, which included performing an operation and conducting a clinical visit. The examination was conducted by two German pediatric surgeons. In addition to this local examination, to demonstrate that the training meets the required African standards, the fellows were also required to pass the COSECSA board examination.

## 3. Results

Nine experienced German pediatric surgeons traveled to Jimma. One colleague came twice, and two colleagues came three times. In total, there were 14 visits. Seven (77.8%) had prior experience with medical stays in developing countries. Thirteen (93%) completed their experience reports. 

In response to the question “Considering your experience, how well were you prepared for Jimma Hospital?”, all 13 rated the preparatory workshops as “good” or “very good” (1.3). The question about the support on-site (“How would you evaluate the support you received during your mission?”) was also answered predominantly with a “good” or a “very good” (1.7). Only one colleague who had been harassed during the stay answered the question with “satisfactory”.

The overall cooperation between the universities (“How well were you able to perform in your role as assistant rather than lead surgeon in Jimma?”) was also rated mainly positive—1.6. Only one colleague gave “satisfactory” rating. He complained that communication and cooperation with the nurses were inadequate. He also complained there was no responsible neonatologist in the Neonatal Intensive Care Unit (NICU). Two colleagues did not comment. 

The success of sharing knowledge (“How successful were you in passing on your experience to your successor and on your ideas of how to improve the program?”) was rated as 1.5. One colleague rated this only as “sufficient”, adding that the Ethiopian colleagues were already “very well versed in surgical techniques. JimmaChild should place more emphasis on the organizational structure of the clinic and the training of nursing staff in the further course of the project”.

The general support by the JimmaChild project team (“How would you rate the overall support provided by the JimmaChild project team of the Dr. von Hauner Children’s Hospital?”) was perceived as very positive (1.2). 

As for the question, “How motivated are you to serve as a multiplicator after your mission in Jimma (e.g., to implement projects and exchanges similar to JimmaChild)?” 11 of the respondents indicated that they were “highly” motivated. 

Within the JimmaChild project, the provision of suitable facilities was a central task of JU. All experience reports indicated that a separate pediatric surgical operating room and a separate pediatric surgical ward were available. In April 2017, however, it was reported that the pediatric surgical ward had been integrated into general surgery without consultation with JimmaChild, and nurses on the ward had been disbanded. After appropriate interventions, this process was reversed. Eleven colleagues reported that the quality of care at the NICU did not meet the requirements.

**Figure 2:**
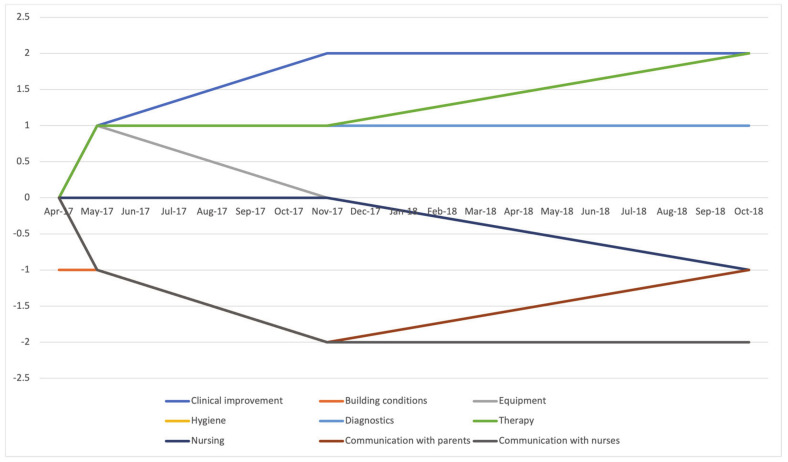
Graphical illustration of the development of individual aspects over time.

The colleagues who traveled to Jimma more than once were asked to provide information over time. While there were mainly improvements in clinical work and therapy, increasing deficits in equipment and communication with the nurses (Figure 2) were reported.

In total, 850 elective and 1800 emergency operations were performed. These included pediatric surgical index operations such as hypospadia repair (*n* = 40), pull-through operations for anorectal malformations (*n* = 31), and Hirschsprung disease (*n* = 33). The proportion of elective operations assisted by German colleagues was 82%.

In June 2019, both fellows passed their final exams with 81% and 84%, respectively. (Broken down, these scores were 23 and 21 for progressive assessments, 12 and 15 for the written examination, and 46 and 47 for the clinical examination.).

## 4. Discussion

In their 2030 Agenda for Sustainable Development, the United Nations (UN) defined good health and well-being as one of the 17 Sustainable Development Goals (SDGs) [4]. This goal was central for Ethiopia, too, and scientific exchanges with other countries have been forged to develop health care. Expenditures for the medical sector have increased more than fourfold since 2005 (USD28 per capita for 2016 [5]). This shows the efforts Ethiopia are making. Particular importance is attached to child health. This is because Ethiopia has also faced and continues to face the problems that Chirdan et al. identified in their work [3]. The care of sick children was hardly available in Ethiopia despite almost half of all Ethiopians being younger than 15. This gap was highlighted in 2018 by Derbew et al. [6]. To address the problem, Ethiopia is taking a somewhat different approach than that described by Ozgediz et al. [7]. The establishment of pediatric surgical care is seen as a proprietary task that should be realized with international cooperation but not through external help. In this context, JU requested the Department of Pediatric Surgery at Dr. von Hauner Children’s Hospital in 2014 to work together on a project to improve the situation for surgically ill children in the Jimma region. JimmaChild’s approach was to provide professional support to the JU in its efforts to establish a pediatric surgery unit and to provide on-site fellowship training. Thus, the approach went much further than that of Calisti et al. [8]. On site, surgery was not only performed, but two fellows were given explicit training to become pediatric surgeons. JimmaChild thus represents a small building block toward improved health care in Ethiopia.

**Figure 3:**
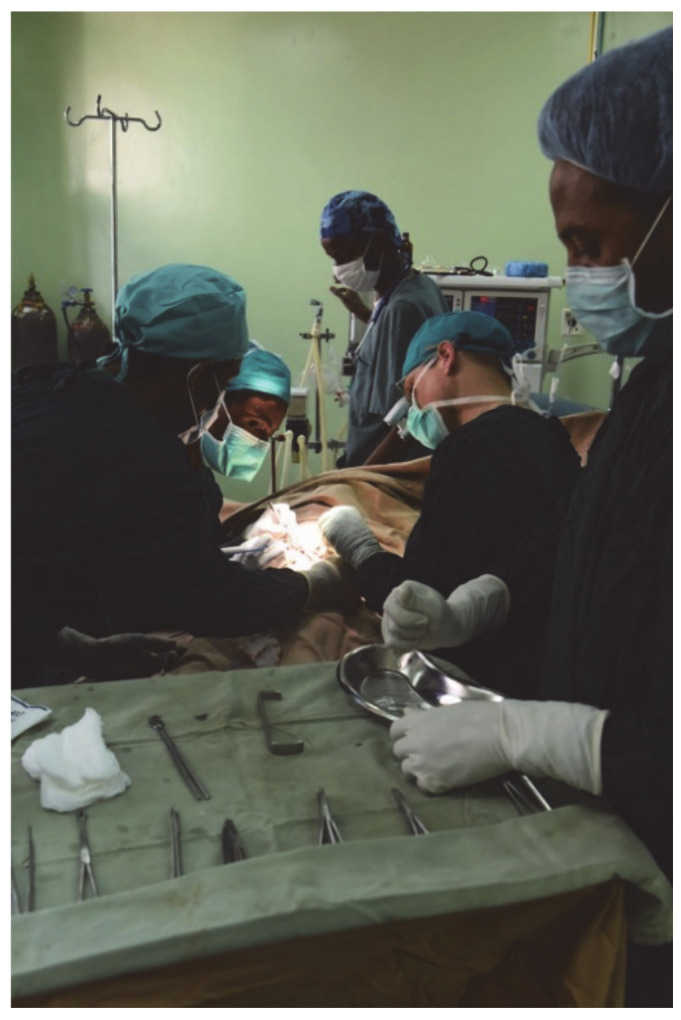
View into the operating room during an operation. In the foreground is the surgical equipment required to perform these operations.

**Figure 4:**
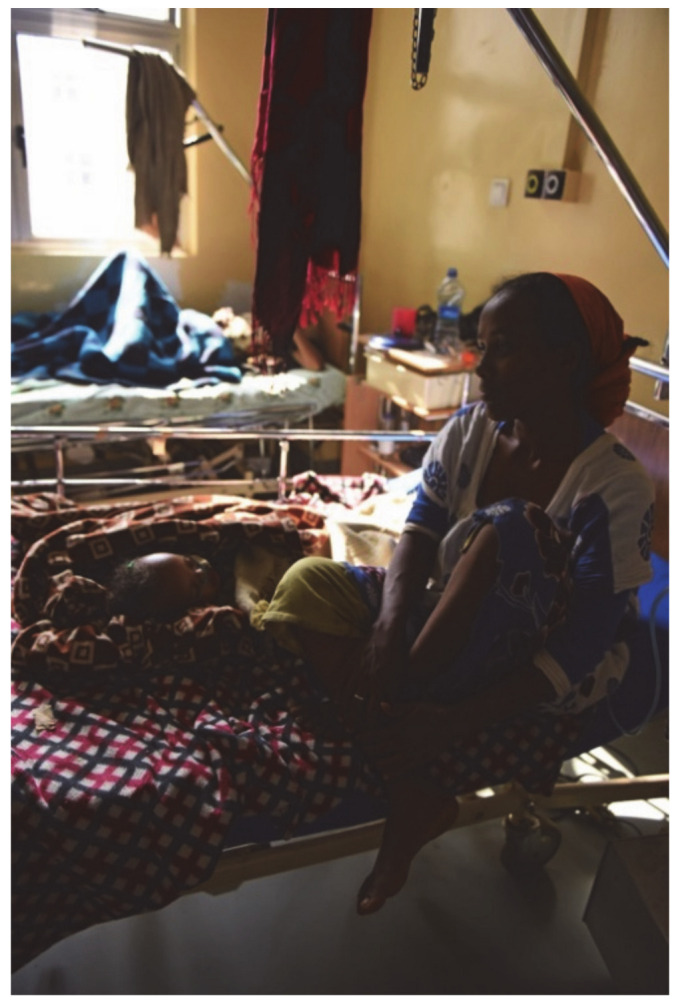
One of the hospital beds in the pediatric surgery ward. The children usually lie in bed together with their mothers, who then also take over the essential part of the care during the inpatient stay.

JU now has its own pediatric surgical unit with its own operating room (Figure 3), ward (Figure 4), and assigned healthcare professionals. Two fellows were successfully trained as pediatric surgeons according to the newly implemented curriculum, allowing them to train specialists independently. 

Here, we will critically discuss what has been a decisive factor in the success of the project, but also which factors still pose considerable challenges.

Since the idea of having a pediatric surgical unit at JU originally came from Ethiopian colleagues, there was great motivation on the Ethiopian side to make the project a success. The newly established curriculum and the overall project plan were well integrated into the existing structures at JU. Through the selection of only very experienced German pediatric surgeons and providing them with intensive preparation, a high degree of knowledge transfer was achieved. On site, the Ethiopian fellows made enormous progress throughout the training. In fact, the decision to conduct the training in Jimma and not in Germany helped them learn to treat even complex cases using limited resources. 

Thanks to the external monitoring, we were able to identify problems at an early stage and to respond accordingly. For example, the administration of JU merged the pediatric surgical ward with the general surgery and dissolved the pediatric surgery care team. As a result of the external monitoring, this matter was identified, and it was possible to intervene with the administration. As problems were detected, they were discussed with the Ethiopians, a solution was devised jointly, and the Ethiopian surgeons were responsible for implementation. Through this approach, we saw that the Ethiopian colleagues recognized and accepted their responsibility for the success of the project.

In addition to many wonderful successes, problems also crystallized. First, there is a problem with acquiring adequate supplies. There is no functioning infrastructure to procure suitable materials in a reasonable time frame—a problem that health systems are struggling with in many African countries [3]. At the start of the project, three instrument trays were purchased using donated funds. In the course of the project, these showed considerable wear and tear. However, such purchases with donated funds tend to worsen the situation as they hinder the development of an infrastructure. As long as external donors provide materials and equipment below the market price or even free of charge, there is no need to establish an independent infrastructure for the purchase and maintenance of medical supplies. Furthermore, local suppliers cannot operate economically compared to the very cheap or free donations, so that there is no market for within the country. As soon as the external donors stop their activities, a supply gap arises that cannot be closed so easily. These connections are described in detail by Dambisa Moyo in her book, Dead Aid [8]. As a consequence, no further purchases were made with donations. Instead, an attempt was made to initiate the development of supplies on-site, which proved to be extremely difficult. For the same reason, JimmaChild did not pay any incentives, which cause similar effects as donated purchases. 

Next, there are challenges with the training of and communication with nurses. This was a problem expressed several times, especially by the colleagues who traveled to Jimma repeatedly. Similar experiences were also described by Calisti et al. [8]. It has become apparent that much more attention should be paid to the education of the entire team. Accordingly, the topic of nurse training became a central component of the follow-up project, EthioChild, which was fortunately granted. It is now planned that the teams traveling for training purposes will always be staffed with nurses. It is also planned that Ethiopian nurses will complete observerships in Germany.

**Figure 5:**
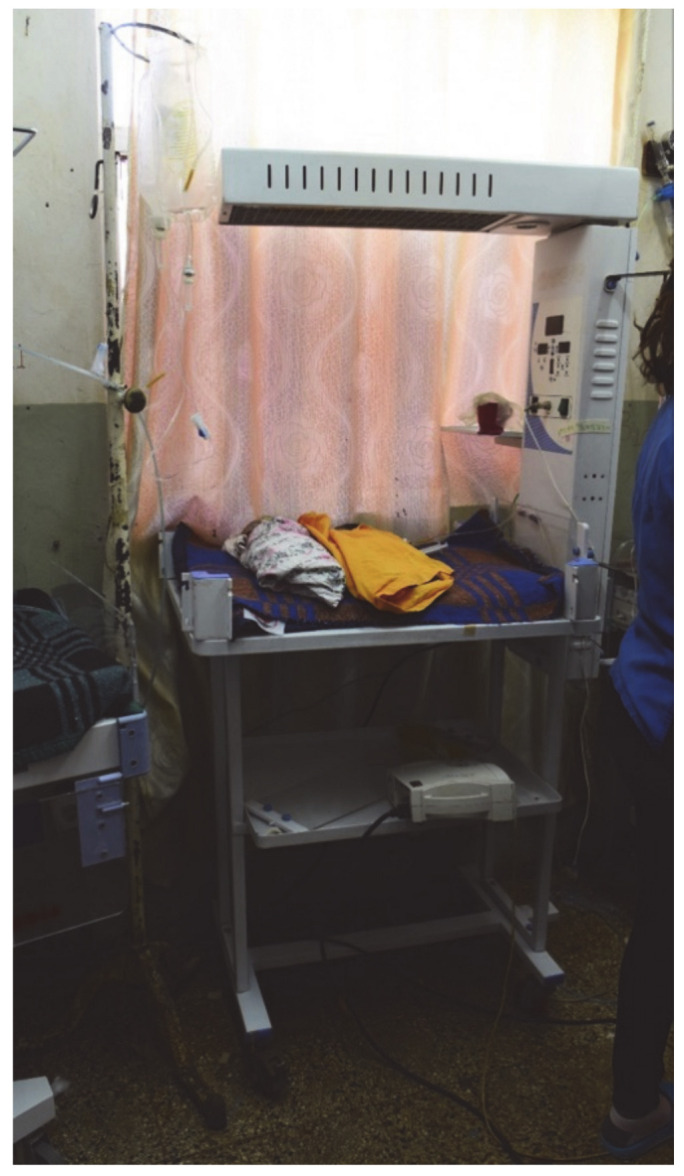
One of the heated beds at the Neonatal Intensive Care Unit (NICU). Due to lack of space, the beds are usually occupied by up to four neonates. From a surgical point of view, a major problem at the NICU is that parenteral nutrition is not available for the children.

Finally, the conditions in the NICU (Figure 5) are a major problem, similar to other African countries [3]. 

Above all, the inadequate structure in the ward and a lack of parenteral nutrition are major limitations that should be addressed by JU.

## 5. Conclusions

The goal of JimmaChild was to establish a pediatric surgery at JU with its own resources and trained specialists. This goal was achieved. We see the success of JimmaChild in the fact that the idea for the establishment of a pediatric surgical unit in Jimma came from the Ethiopian surgeons who were then driven to contribute their ideas to the development of this project. The responsibility for the success of JimmaChild was always with the Ethiopians, and they fully accepted it.

Thanks to the thorough preparation of the experienced German pediatric surgeons, they were able to carry out their tasks on-site and a high degree of knowledge transfer was achieved. External monitoring made it possible to identify and respond to problems in the program flow at short notice.

## Data Availability

The data presented here can be requested from the corresponding author.

## Supplementary Materials

The following are available online at https://www.mdpi.com/2227-9067/8/3/244/s1, File S1: Pediatric Surgical Curriculum; File S2: Erfahrungsbericht JimmaChild über den Einsatz in Jimma XXX.

## Abbreviations

JU	Jimma University
LMU	Ludwig-Maximilians University
COSECSA	College of Surgeons of East, Central, and Southern Africa
DGKCH	German Society for Pediatric Surgery
UN	United Nations
SDG	Sustainable Development Goals
